# The Potential of Mesenchymal Stem Cell-Derived Exosomes in Cardiac Repair

**DOI:** 10.3390/ijms252413494

**Published:** 2024-12-17

**Authors:** Dipan Kundu, Song Yi Shin, William M. Chilian, Feng Dong

**Affiliations:** Department of Integrative Medical Sciences, Northeast Ohio Medical University, Rootstown, OH 44272, USA; dkundu@neomed.edu (D.K.); sshin@neomed.edu (S.Y.S.); wchilian@neomed.edu (W.M.C.)

**Keywords:** exosome, stem cells, angiogenesis, apoptosis, autophagy, inflammation, cardiac repair, microRNA

## Abstract

Cardiovascular diseases (CVDs) are the leading cause of death worldwide, and effectively repairing the heart following myocardial injuries remains a significant challenge. Research has increasingly shown that exosomes derived from mesenchymal stem cells (MSC-Exo) can ameliorate myocardial injuries and improve outcomes after such injuries. The therapeutic benefits of MSC-Exo are largely due to their capacity to deliver specific cargo, including microRNAs and proteins. MSC-Exo can modulate various signaling pathways and provide several beneficial effects, including cytoprotection, inflammation modulation, and angiogenesis promotion to help repair the damaged myocardium. In this review, we summarize the cardioprotective effects of MSC-Exo in myocardial injury, the underlying molecular mechanism involved in the process, and various approaches studied to enhance their efficacy based on recent findings.

## 1. Introduction

Despite significant advancement in the past years, myocardial injury, in particular myocardial infarction (MI), still remains one of the leading causes of morbidity and mortality worldwide [1,2,3]. There is an unmet need for therapeutic intervention to repair damaged heart tissue following myocardial injuries. The repair is a finely coordinated complex process involving resolution of inflammation, neovascularization, and restoration of lost cardiomyocytes.

Stem cell-based therapy, especially mesenchymal stem cell (MSC), has been one of the most studied therapies for cardiac repair and regeneration. MSCs are multipotent adult stem cells that can be isolated from various sources, including bone marrow (BM), adipose tissues, and umbilical cord [4,5]. Numerous preclinical and clinical studies have shown the beneficial effects of MSCs on myocardial injury. However, despite having tremendous potential, MSC-based therapy yielded only modest clinical benefits. Several factors have been identified as the cause of this limited efficacy, such as poor retention and survival of MSCs and insufficient homing to the site of injury. MSC transplantation may also induce adverse effects, such as an immune response and malignancy [6,7,8,9,10,11]. More studies have demonstrated that the therapeutic effects of MSCs on cardiac repair are mainly exerted by their paracrine effects, particularly via secretion of extracellular vesicles (EV) [12,13,14,15,16]. EVs are a heterogeneous group of cell-secreted, nano-sized particles and are classified mainly based on their size, such as exosomes (30–150 nm), microvesicles (100–1000 nm), and apoptotic bodies (100–5000 nm) [17,18,19]. Exosomes and EVs are sometimes used interchangeably in the literature. While we mainly discuss exosomes in this review, studies on EVs are also presented since they primarily represent the exosome population in those particular studies. Exosomes are released and received by almost all cells in the body. They carry various genetic cargo closely related to their origin and function, including proteins, lipids, and RNAs. Exosomes can transfer from their originating cell to various distant parts of the body through systemic circulation, where they can deliver their cargo [20,21,22,23,24,25]. The ability of exosomes to mediate cellular communication makes them important vectors for delivering a variety of “drugs” throughout the body.

Recently, the focus of research has been shifted from using MSC-based transplantation therapy to secretory exosome therapy. Exosomes derived from MSCs (MSC-Exo) hold several advantages over other stem cell therapies. (1) MSCs can modulate immune responses, making them highly beneficial for treating inflammatory diseases; (2) MSC-Exo contains bioactive molecules like proteins, lipids, RNAs, and growth factors that can promote tissue repair and regeneration without the complications associated with whole stem cell therapies. (3) The very small size allows MSC-Exo to easily pass through different biological barriers, including capillaries, and transport various contents to virtually any tissue site, eliminating one of the drawbacks associated with MSC-based therapy. (4), MSC-Exo are less likely to lead to tumor formation compared to stem cell therapy, making them a safer therapeutic option [26,27,28,29,30]. (5) Stem cell therapies, particularly those using embryonic stem cells, face significant ethical challenges due to the need for harvesting cells from embryos. MSCs can be obtained from adult tissues such as bone marrow and adipose tissue; therefore, MSC-Exo can avoid these ethical concerns. (6) MSC-Exo are easier to produce and store than live stem cells. (7) MSC-Exo has been shown to be effective in a wide range of therapeutic areas, including cardiovascular diseases. (8), MSC-Exo can serve as a natural drug delivery carrier that can be manipulated to enhance their therapeutic effects. Due to these advantages, MSC-Exo is emerging as a promising alternative to traditional stem cell therapies, providing a safer, more efficient, and ethically acceptable option for therapeutic interventions [31,32].

However, their clinical application must address several issues, including their production, delivery, and rapid clearance in vivo. As a result, researchers have been continuously studying new approaches to enhance the efficacy and stability of MSC-Exo. In this review, we will summarize research that highlights the cardioprotective effects of MSC-Exo after myocardial injury and their molecular mechanisms. We will also discuss recent advancements in addressing the limitations and enhancing the therapeutic efficacy of MSC-Exo in cardiac repair.

## 2. The Beneficial Role and Mechanisms of MSC-Exo in Cardiac Repair

**Figure 1:**
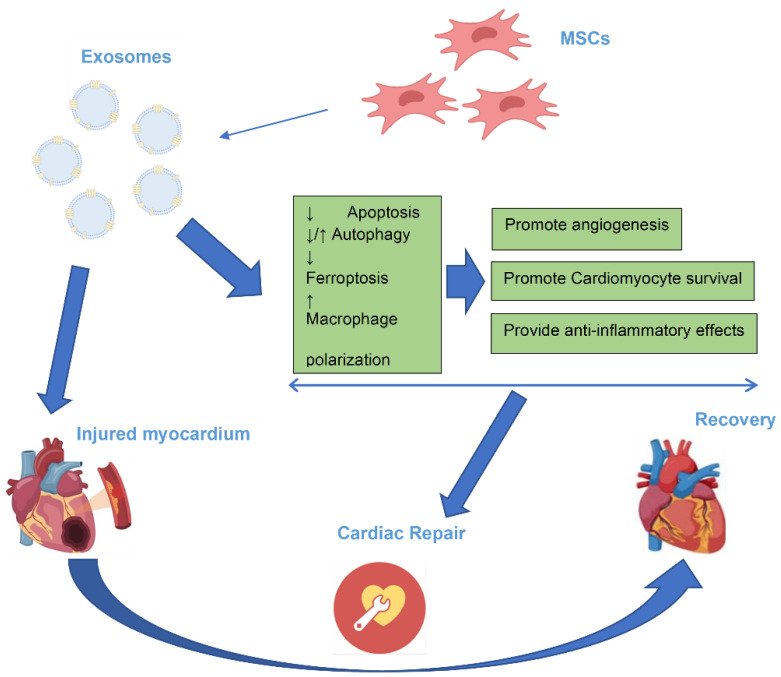
The beneficial role and mechanisms of MSC-Exo in cardiac repair.

Growing evidence suggests that MSC-Exo may accumulate in the ischemic myocardial tissue and regulate cell proliferation, apoptosis, autophagy, inflammation, and angiogenesis during tissue repair. Additionally, they have been used as a drug delivery system by being loaded with a specific protein or microRNA to specifically target the injured myocardium. They can modulate the expression of important cellular signaling molecules, including regulatory proteins, and influence various signaling pathways in cells, including cardiomyocytes and macrophages, to alleviate myocardial injury and improve cardiac function (Figure 1, Table 1).

### 2.1. MSC-Exo Protect Cardiomyocytes in Myocardial Injury

#### 2.1.1. MSC-Exo in Cardiomyocyte Apoptosis

Apoptosis of cardiomyocytes is a significant factor in conditions such as heart attack and heart failure, contributing to myocardial loss. Increasing evidence suggests that MSC-Exo can improve cardiac function by protecting against cardiomyocyte apoptosis during myocardial injury. The administration of MSC-Exo through intravenous, intramyocardial, or intrapericardial sac routes has all been shown to reduce the apoptosis of native cardiomyocytes in different cardiac injuries through various anti-apoptotic pathways [33,34,46,54,59,60,66]. Cui et al. showed that adipose-derived MSC-Exo (ADMSCs-Exo) effectively reduced apoptosis and protected ischemic myocardium from ischemia/reperfusion (I/R) injury in rats through activation of the Wnt/β-catenin signaling pathway [46]. In another study, extracellular vesicles (EV) derived from MSC (MSC-EV) improved cardiomyocyte viability in MI injury by targeting zinc finger antisense 1 (ZFAS1) or the Akt/Nrf2/HO-1 pathway [33]. Chen et al. demonstrated the effectiveness of MSC-Exo in the transverse aortic constriction (TAC) model of pressure overload-induced heart failure mice. Intramyocardial injection of MSCs-Exo significantly protected myocardium against cardiac hypertrophy, attenuated myocardial apoptosis, and preserved heart function in TAC injury [54]. In a more recent study by Rocca et al. in 2024, it was demonstrated that EVs derived from human gingival mesenchymal stem cells (hGMSCs) played a protective role for HL-1 cardiomyocytes under acute hypoxic conditions. The study investigated the expression of various inflammatory, cell survival, and apoptotic markers, including the pro-apoptotic proteins CASP3 and BAX, and showed that the EVs reduced apoptosis and enhanced cell survival [66].

Endoplasmic reticulum (ER) stress occurs when the capacity of the ER to fold proteins becomes saturated, and ER stress-induced apoptosis is also implicated in the pathogenesis of myocardial I/R injury. Treatment with MSC-EV inhibited ER stress and increased survival in H9c2 cells exposed to I/R by activating the PI3K/Akt pathway [60].

A few studies have shown that MSC-Exo carries important constituent molecules in their cargo that can play a crucial role in apoptosis and cardioprotection. Patil et al. demonstrated an interesting mechanism by which MSC-Exo provides protective action after myocardial ischemic injury [34]. They showed that MSC-Exo contains MFGE8, a glycoprotein that bridges externalized phosphatidylserine (PS) on the apoptotic cell surface to alphaVbeta3 or alphaVbeta5 integrins on the phagocyte. MFGE8 enhances the process of opsonization of dead cells and also activates phagocytic signaling. This leads to increased phagocytosis of apoptotic cells, resolution of inflammation, and efficient cardiac recovery after injury [34]. The work of Li et al. revealed that bone marrow MSC-Exo contains itchy E3 ubiquitin ligase (ITCH), which played a key role in the inhibition of apoptosis of rat cardiomyoblasts by facilitating ASK1 ubiquitination, leading to improved myocardial injury in acute MI [59].

#### 2.1.2. MSC-Exo in Cardiomyocyte Autophagy

Autophagy is an essential metabolic process that degrades aged or damaged proteins and organelles into amino acids and fatty acids for recycling and energy production, and it maintains the homeostasis of the intracellular microenvironment. Usually, nutrient deficiency or metabolic stress triggers autophagy, but it can also be activated by myocardial injury. Activation of autophagic flux in the heart can be beneficial or maladaptive, depending on the context [67,68,69]. Autophagy can inhibit the inflammatory response by removing invading pathogens and damaged mitochondria. In addition, autophagy may enhance the clearance of apoptotic and necrotic cells to promote repair after myocardial injury. On the other hand, excessive activation of autophagy can have detrimental effects and aggravate tissue damage, a more common scenario following myocardial injury [67,69]. Xiao et al. showed that transplanted MSCs improved myocardial function and reduced infarct size by reducing autophagic flux in the infarcted myocardium of mice. The reduction of autophagic flux and other beneficial effects of transplanted MSC were mediated through secreted exosomes since pretreatment of MSC with the exosomal inhibitor GW486 abolished the effects [38]. Liu et al. observed an increase in the expression of inflammatory cytokines and miR-93-5p following acute MI (AMI) in both patients and animal models [39]. Exosomes containing miR-93-5p, derived from adipose-derived stem cells (ADSC), significantly improved infarction-induced myocardial damage by suppressing autophagy and inflammation [39]. In vitro, activating autophagy indirectly increased the expression of inflammatory cytokines; however, miR-93-5p expression greatly decreased autophagy and inflammatory cytokine expression caused by hypoxia. This suggests that miR-93-5p-containing MSC-Exo’s beneficial effects were due to their ability to lower autophagy [39]. Another study showed that injecting exosomes containing miR-301 from BMSCs not only helped maintain cardiac function but also reduced the number of autophagosomes, resulting in a decreased LC3-II/LC3-I ratio in MI rats, indicating a suppression of autophagy in the heart [40]. MSC-Exo modulates different autophagy signaling pathways, such as MEKK1-MKK4-JNK, CHK2-Beclin2, and miR-143/Bcl-2/Beclin-1, to inhibit autophagy and alleviate H/R injury-induced cardiomyocyte damage [47,48,70]. In their study, Chen et al. showed that an autophagy activator canceled out the beneficial effects of MSC-Exo, while an autophagy inhibitor further enhanced the effects. This shows that MSC-Exo protects against H/R damage by inhibiting autophagy [48].

Although most studies have suggested that MSC-Exo suppresses prevailing hyper-autophagy following myocardial injury to exert beneficial effects, a few studies have also shown that induction of autophagy is necessary for MSC-Exo-mediated cardioprotection. A study by Liu et al. has demonstrated that MSC-Exo reduced H2O2-induced ROS production and cell apoptosis by inducing cardiomyocyte autophagy via AMPK/mTOR and Akt/mTOR pathways. In vivo, MSC-Exo injection in rats with I/R injury significantly reduced the size of the myocardial infarction and improved heart function by increasing myocardial autophagy [49]. Similarly, Zou et al. also showed that MSC-Exo protected against MI injury by promoting autophagy [71].

#### 2.1.3. MSC-Exo in Cardiomyocyte Ferroptosis

Ferroptosis is an intracellular iron-dependent form of cell death that is distinct from apoptosis and autophagy. It is a relatively newly discovered form of cell death that is featured in a wide range of diseases, such as cardiovascular disease, neurological disease, liver disease, and cancer. Recently, more and more studies have reported that MSC-Exo can modulate ferroptosis in different cellular injuries, including liver injury, lung injury, and spinal cord injury [72,73,74,75,76].

The effects of MSC-Exo in cardiomyocyte ferroptosis have not been well-studied. Evidence suggests that ferroptosis occurs in the infarcted myocardium and in cardiomyocytes following hypoxia-induced injury [77,78]. One study reported that intramyocardial injection of MSC-Exo inhibited ferroptosis and reduced myocardial injury by delivering miR-23a-3p, which suppresses divalent metal transporter 1 (DMT1) expression, a key mediator of ferroptosis [41]. A recent study reported that MSC-Exo suppressed H/R-induced cardiomyocytes’ ferroptosis by upregulating miR-330-3p, which regulates the BAP1/SLC7A11/IP3R axis to inhibit mitochondrial permeability transition pore (mPTP) opening and mitochondrial dysfunction [79]. Similarly, another study reported that MSC-Exo treatment attenuated I/R-induced cardiac injury by inhibiting cardiomyocyte ferroptosis by modulating the Pum2/PRDX6 axis, thereby ameliorating cardiac function [80]. DOX-induced cardiotoxicity involves ferroptosis in its pathological progression. Yu et al. showed that MSC-Exo could suppress ferroptosis in DOX-induced cardiotoxicity [81]. Given the increasing evidence of ferroptosis in different myocardial injuries, further study is warranted to explore the role of MSC-Exo in cardiomyocyte ferroptosis.

#### 2.1.4. MSC-Exo Protects Cardiomyocytes Against Infection and Drug-Induced Myocardial Injury

Myocardial injury is a common complication in different systemic inflammations, and some pharmacologically active compounds may also lead to myocardial damage. MSC-Exo has been shown to be effective in providing cardioprotection against this condition. Kore et al. found that MSC-Exo carries proteins that can protect cardiomyocytes from lipopolysaccharide (LPS)-induced cell death and promote cell survival [82]. Zhou et al. studied sepsis-induced myocardial injury and found that exosomes derived from human umbilical cord MSC (hucMSC-Exo) transferred Pink1 mRNA to recipient cardiomyocytes, leading to the restoration of mitochondrial function and cardiac recovery in sepsis [55]. Another study highlighted the role of MSC exosomal circRTN4 in preventing sepsis-induced myocardial injury by regulating the circRTN4/miR-497-5p/MG53 pathway, further supporting the cardioprotective role of MSC-Exo in sepsis [37]. Research by Shi et al. revealed that hucMSC-Exo could promote the transformation of fibroblasts into myofibroblasts in inflammatory environments, reducing cardiomyocyte apoptosis [36]. HucMSC-Exo also alleviated virus-induced myocarditis by activating the AMPK/mTOR-mediated autophagy pathway to reduce cardiomyocyte apoptosis [56]. Treatment with the anticancer drug doxorubicin (DOX) can induce cardiotoxicity, significantly impacting patient survival. In a study by Zhong et al., it was demonstrated that MSC-EV inhibited DOX-induced reactive oxygen species (ROS) and apoptosis through the miR-100-5p/NOX4 pathway to protect cardiomyocytes [61]. Similarly, another study reported that adipose-derived MSC-Exo alleviated cyclophosphamide (CYP)-induced cardiotoxicity in rats by suppressing oxidative stress, apoptosis, and autophagy [57].

#### 2.1.5. Specific microRNAs Play an Important Role in the Cardioprotective Effects of MSC-Exo

MicroRNAs present in MSC-Exo/EV are crucial in delivering the cardioprotective effects. A recent study by Wang et al. showed that by delivering miR-144-3p into the I/H cardiomyocytes, MSC-EV suppressed Rho-kinase 1 (ROCK1) and stimulated the PI3K/AKT/mTOR pathway, thus inhibiting ischemia/hypoxia (I/H)-induced cardiomyocyte apoptosis and autophagy [62]. Other studies have also confirmed that MSC-Exo/EV can deliver specific miRNAs, such as miR-150-5p, miR-149, miR-486-5p, miR-183-5p, and miR-19a, to cardiomyocytes in cardiac injuries, which in turn activate different signaling pathways to provide cardioprotective actions, including inhibition of apoptosis, autophagy, oxidative stress, and myocardial remodeling [45,51,52,53,63].

Knockdown or downregulation of specific microRNA has been shown to abolish the cardioprotective effects of MSC-Exo, highlighting the importance of their presence in the exosomes. Zhu et al. showed that intravenous administration of bone marrow MSC-Exo exerted marked cardioprotective effects in post-MI mice by suppressing the expression of the proapoptotic genes *p53* and *BAK1* in cardiomyocytes. However, miR-125b knockdown in exosomes lost the capability to suppress them, suggesting that miR-125b-5p plays a critical role in facilitating ischemic cardiac repair mediated by MSC-Exo [42]. Similarly, other microRNAs, such as miR-214 and miR-24, have been reported as the key mediators in protecting cardiomyocytes from apoptosis both in the in vitro and in vivo MI animal model [44,83].

Other studies have taken different approaches to demonstrate the importance of specific microRNAs in the MSC-Exo. Cheng et al. showed that intramyocardial injection of miR-210-carrying MSC-Exo reduced infarct size after coronary ligation in rats. Their study demonstrated that overexpression of miR-210 in cardiomyocytes activated identical signaling pathways with similar cellular effects compared to endogenous exosomal miR-210 activity, suggesting that the presence of miR-210 primarily contributes to the beneficial effects of exosomes [43]. MSC-Exo was also effective in atrial fibrillation (AF) in a study that demonstrated miR-148a in the MSC-Exo played a vital role in inhibiting SMOC2 to provide cardioprotective effects. The study also showed that the miR-148a mimic was able to mediate similar beneficial effects, highlighting the key role of miR-148 in MSC-Exo-mediated cardioprotection [58].

Most studies focusing on microRNAs have reported that upregulation of certain microRNAs, via MSC-Exo, orchestrates the cardioprotective effects in injuries. In contrast, some microRNAs have been shown to have a negative role in cardioprotection, and thus their downregulation by MSC-Exo treatment has resulted in the desired therapeutic effects. For example, Yu et al. demonstrated that sponging miR-556-5p was necessary to reduce apoptosis and oxidative stress via long non-coding RNA alpha-2-macroglobulin antisense RNA 1 (Lnc A2M-AS1) in the MSC-Exo [64]. In another study, the expression of miR-17-5p was elevated in response to hypoxia/reoxygenation (H/R) injury, and this was reversed by treatment with MSC-Exo, leading to improved cell survival and reduced cell apoptosis [65]. The work of Li et al. showed that MSC-Exo upregulated HCP5 expression, which in turn could sponge miR-497 to disinhibit the IGF1/PI3K/AKT pathway in protecting cardiomyocytes against H/R injury [50]. Collectively, these studies highlight the importance of specific microRNAs modulation by MSC-Exo in providing cardioprotection during injury.

#### 2.1.6. MSC-Exo Modulate Specific Signaling Pathways to Mediate Cardiac Repair

MSC-Exo can deliver specific cargo to modulate specific signaling pathways to facilitate cardiac repair. For example, Cheng et al. demonstrated the endocytosis of miR-210-containing MSC-Exo into cardiomyocytes, while the immunofluorescence study confirmed the co-localization of miR-210 within exosomes [43]. miR-210 targeted AIFM3 to regulate the PI3K/AKT and p53 signaling. MiR-210 mimic-transfected cardiomyocytes showed downregulation of AIFM3, p-AKT, and p-PI3K, while knockdown of miR-210 abolished this, suggesting a key role of miR-210 within MSC-Exo in regulating these signaling pathways. Downregulation of PI3K/AKT and p53 signaling led to inhibition of apoptosis and increased cellular survival in MI mice [43]. Similarly, Wang et al. showed that MSC-Exo delivered miR-144-3p into ischemia-hypoxia (I/H) cardiomyocytes, and miR-144-3p targeted ROCK1 to stimulate the PI3K/AKT/mTOR pathway to protect cardiomyocytes from I/H injury [62]. Another study showed that MSC-Exo activated AMPK/mTOR signaling to mediate the autophagy flux pathway in alleviating CVB3 virus-induced myocarditis [56]. Both in vivo and in vitro studies confirmed that MSC-Exo significantly increased the levels of pAMPK but decreased the level of pmTOR. Activation of AMPK/mTOR signaling was associated with increased autophagy flux and apoptosis attenuation to improve cardiac function [56]. Together, these studies highlight the importance of turning on a specific signaling pathway for MSC-Exo-mediated cardiac repair. 

### 2.2. MSC-Exo Ameliorate Inflammation in Myocardial Injury

#### 2.2.1. MSC-Exo in Inflammation

MSC-Exo are well-recognized immunomodulating agents. Multiple studies have demonstrated their anti-inflammatory effects, promoting cardiac repair (Table 2). In MI-induced myocardial injury, MSC-Exo has been shown to decrease inflammation and improve cardiac function. These studies suggest that the anti-inflammatory effects are mediated through different mechanisms involving different signaling pathways [84,85,86,87]. Yan et al. revealed that MSC-Exo could suppress inflammation and fibrosis in cardiomyocytes by inhibiting the NF-κB signaling pathway through the miR-5p/TRAF axis [87]. Teng et al. studied the effect of MSC-Exo on T-lymphocyte proliferation and reported that exosome treatment had an inhibitory proliferative effect in vitro, suggesting inhibition of inflammation [84].

Pyroptosis, or caspase 1-dependent cell death, is inherently inflammatory and is triggered by various pathological stimuli, such as stroke, heart attack, or cancer. Tang et al. showed that MSC-Exo protected the myocardium against ischemia/reperfusion injury through inhibition of pyroptosis by targeting NLRP3 inflammasome signaling [88]. Additionally, a study by Pan et al. demonstrated that MSC-EV could transfer miR-223-3p to cardiomyocytes to repress LPS-induced cardiomyocyte inflammation and pyroptosis by disrupting the FOXO3/NLRP3 axis, leading to the alleviation of cardiac dysfunction [89].

Specific miRNAs have also been modulated as a key factor in the anti-inflammatory action provided by MSC-Exo. Wang et al. found that MSC-Exo is enriched with miR-223, which could be delivered to cardiomyocytes to confer cardioprotection in sepsis by downregulating target proteins Sema3A and Stat3. They also reported that ablation of miR-223 in MSCs can reprogram the protein contents of exosomes, which abrogates the anti-inflammatory and cardioprotective effects of exosomes [90]. Other studies also demonstrated the importance of specific miRNAs in the MSC-Exo to repress inflammation and myocardial injury [86,91]. In a study conducted by Shao et al., the effects of MSC-Exo and MSC were compared. It was found that MSC-Exo when intramyocardially administered after acute MI in rats, provided significantly better anti-inflammatory effects and improved cardiac function compared to MSC injection. The study revealed that while MSC-Exo and MSCs had similar miRNA expression profiles, the expression of several miRNAs from MSC-Exo significantly differed from that of MSCs. The authors suggested that this difference in miRNA expression could be the reason behind the superior therapeutic effects of MSC-Exo compared to MSCs [92].

**Table 2:** MSC-Exo provides anti-inflammatory effects following myocardial injury.

Model of Myocardial Injury	MSC-Exo/EV Source	Dose	Administration	Mechanisms/Signaling Pathway	Related miRNA	Effects	Author
Myocardial Infarction (MI)							
Mice MI by LAD ligation		100 μg	Intracardiac injection after 24 h of MI induction	Suppress expression of Bcl-2– like protein 11	miR-200b-3p	Reduce apoptosis of cardiomyocytes, myocardial fibrosis, and inflammation in MI mice; improve cardiac function	Wan et al. (2022) [93]
Rats MI by LAD ligation	Rat bone marrow	80 μg	Intramyocardial	Impair T-lymphocyte proliferation		Promote angiogenesis, decrease inflammation, and preserve cardiac function	Teng et al. (2015) [84]
Mice with heart failure (HF) by permanent LAD ligation	Mouse bone marrow	5 μg once a week for three times	Intravenous (Tail-vein)	Inhibit NF-κB signaling pathway through miR-129-5p/TRAF3	miR-129-5p	Suppress oxidative stress, apoptosis, inflammation, and fibrosis in cardiomyocytes in mice with HF; alleviate ventricular dysfunction	Yan et al. (2022) [87]
Mice MI by LAD ligation	Mouse bone marrow	10 μg	Intravenous (Tail-vein)	Downregulate BCL6/MD2/ NF-κB signaling axis	miR-302d-3p	Repress inflammation and cardiac remodeling after MI	Liu et al. (2022) [86]
Rats MI by LAD ligation	Human umbilical cord		Intrapericardial	Induce regulatory T-cell differentiation and activate PP2A/p-Akt/Foxo3 pathway		Inhibit inflammation and promote cardiac repair following MI	Zhu et al. (2022) [94]
Mice MI by LAD ligation	Human PMSCs		Intravenous (Tail-vein)	Modulate gut microbiota to suppress inflammation		Suppress inflammation and ameliorate myocardial fibrosis and left ventricular remodeling after MI	Yang et al. (2022) [95]
Rats MI by LAD ligation	Rat bone marrow	20 μg	Intramyocardial	Modulate the expression miRNAs related to cardiac repair		Inhibit cardiac fibrosis, inflammation, and improve cardiac function	Shao et al. (2017) [92]
Rats MI by LAD ligation	Rat bone marrow	105 μg	Intramyocardial	Reduce expression and activation of Stat1 in macrophages and promote macrophage M2 polarization	miR-139-3p	Alleviate inflammation and promote cardiac repair after MI	Ning et al. (2023) [96]
Mice MI by LAD ligation	Rat bone marrow		Intramyocardial	Suppress the NF-κB signaling pathway and partially activate AKT1/AKT2 signaling to promote macrophage M2 polarization		Reduce post-infarction inflammation and attenuate myocardial injury after MI	Xu et al. (2019) [97]
Mice MI by LAD ligation	Mouse bone marrow	50 μg	Intramyocardial	Suppress the NF-κB signaling pathway and upregulate Nrf2/HO-1 signaling; increase M2 macrophage polarization		Attenuate cardiomyocyte apoptosis, inflammation, and improve cardiac function in MI mice	Ning et al. (2021) [98]
Rats MI by LAD ligation	Human	40 μg	N/A	Upregulate Sirt1 expression by sponging miR-138-5p	miR-138-5p	Decrease apoptosis and pyroptosis, and attenuate MI progression	Mao et al. (2019) [99]
Ischemia- Reperfusion(I/R) injury							
Mice I/R injury by LAD ligation		50 μg	Intramyocardial	Promote macrophage M2 polarization and downregulate TLR4/NF-κB/PI3K/Akt signaling	miR-182	Alleviate inflammation levels in the heart and serum following I/R injury, and improve cardiac function	Zhao et al. (2019) [100]
Mice I/R injury by LAD ligation	Mouse bone marrow	50 μg	Intramyocardial	Promote macrophage M2 polarization	miR-21-5p	Reduce inflammation and attenuate myocardial injury	Shen et al. (2021) [101]
Mice and Swine I/R injury by LAD ligation	Mouse bone marrow	10 μg	Intramyocardial	Promote macrophage M2 polarization by targeting Klf13, Tgfbr1, and Daam1	miR-125a-5p	Attenuate cardiomyocyte apoptosis, cardiac inflammation, and increases angiogenesis	Gao et al. (2023) [102]
Mice I/R injury by LAD ligation	Mouse bone marrow	200 μg	Intravenous	Preferentially accumulate in the heart in injured mice to provide cardio-protection	miR-21	Inhibit apoptosis, alleviate inflammation, and improve cardiac function	Wei et al. (2021) [103]
Miscellaneous injury							
STZ-induced diabetic cardiomyopathy in ApoE−/− mice	Mouse bone marrow	50 μg	Intraperitoneal	Downregulate inflammation associated TAK1-pJNK-NFKB pathway and promote macrophage M2 polarization in heart		Improve cardiac function with reduced cardiac hypertrophy and fibrosis in diabetic cardiomyopathy	Banerjee et al. (2024) [104]
Doxorubicin- induced dilated cardiomyopathy	Mouse bone marrow	300 μg after 7 days of dox induction	Intravenous (Tail-vein)	Promote macrophage M2 polarization through activation of JAK2-STAT6 signaling		Suppress circulatory inflammation response and improve cardiac function in dilated cardiomyopathy	Sun et al. (2018) [105]
LPS-induced myocardial injury	Mouse bone marrow	100 μg/kg body wt.	Intravenous (Tail-vein)	Downregulate FOXO3/NLRP3 signaling	miR-223-3p	Repress LPS-induced cardiomyocyte inflammation, pyroptosis, and cardiac dysfunction	Pan et al. (2022) [89]
Mice sepsis cardiomyopathy induced by cecal ligation and puncture (CLP)	Mouse bone marrow	2 μg/g body wt.	Intravenous	Suppress PTEN expression and enhance the activity of B-Catenin	miR-141	Alleviate CLP-induced inflammatory infiltration in mouse myocardial tissues and ameliorate myocardial injury	Pei et al. (2021) [91]
Rats primary cardiomyocytes with I/R injury in vitro	Human bone marrow			Downregulate expression of NLPR3 inflammasome	miR-320b	Reduce pyroptosis and inflammation in I/R injury	Tang et al. (2020) [88]

Zhu et al. showed that intrapericardially injected MSC-Exo accumulated in the mediastinal lymph node (MLN) and induced regulatory T cell differentiation to promote cardiac repair by Foxo3 activation. Foxo3 regulated inflammatory cytokines (IL-10, IL-33, and IL-34) expression and built up a regulatory T cell (Treg)-inducing niche in the MLN [94]. Interestingly, one study reported that exosomes derived from human placental mesenchymal stem cells (PMSCs-Exo) could modulate gut microbiota to suppress inflammation in MI. The study showed that intravenous injection of exosomes regulated the relative abundances of different microbial populations and reduced the level of pro-inflammatory indicators (IL-1β, IL-6, TNF-α, MCP-1) in plasma and myocardial tissues [95].

Typically, MSC-Exo is administered immediately or shortly after the induction of myocardial injury. Kore et al. took an interesting approach to test whether pretreatment with MSC-Exo before the induction of injury is beneficial in cardiac injury. Their study showed, interestingly, that MSC-Exo pretreatment by intravenous administration 30 min prior to the left coronary artery (LCA) ligation suppressed inflammatory signals during both acute and chronic ischemic injury [106]. Exosome pretreatment improved cardiac function and animal survival after LCA ligation. Treatment modulated proteomic changes both in infarct and peri-infarct areas, especially in the peri-infarct areas, and the anti-inflammatory actions were mainly due to the modulation of lOX-1 expression following injury [106].

#### 2.2.2. Macrophage Polarization by MSC-Exo in Inflammation and the Role of Specific miRNAs

Macrophage polarization is an important property of MSC-Exo in modulating inflammation during cardiac injury. Several studies have shown that MSC-Exo can modify the polarization of proinflammatory M1 macrophages to an anti-inflammatory M2 phenotype, and certain miRNAs play an important role in polarization. Zhao et al. demonstrated that injecting MSC-Exo into the heart tissue after myocardial I/R reduced inflammation levels in both the heart and blood. The study showed that MSC-Exo promoted macrophage polarization both in vivo and in vitro via shuttling miR-182, which interacted with toll-like receptor 4 (TLR4) as a downstream target [100]. Gao et al. demonstrated that miR-125a-5p is enriched in MSC-Exo, and it plays an important role in providing cardioprotective actions [102]. They found that intramyocardial delivery of miR-125a-5p mimic into the heart tissue of mice with ischemia/reperfusion (I/R) injury, as well as administering MSCs or MSC-Exo, resulted in similar protective effects. These interventions improved heart function and reduced adverse remodeling. Their study also revealed that miR-125a-5p mimic treatment alone could increase M2 macrophage polarization and decrease cardiomyocyte apoptosis and inflammation in both mice and porcine without any significant side effects [102]. In another study, inhibition of miR-21-5p in MSC-Exo reduced their polarization capacity, while miR-21-5p mimics promoted the polarization of RAW264.7 cells to the M2 phenotype and decreased inflammatory factors in the culture supernatant, suggesting that miR-21-5p in MSC-Exo is critical for the anti-inflammatory effects [101]. A study by Xu et al. also confirmed macrophage polarization as an important mediator of MSC-Exo’s anti-inflammatory and cardioprotective effects in myocardial injury [97].

The ability of macrophage polarization has also been one of the key attributes of MSC-Exo to mediate cardioprotection during cardiomyopathy in different diseases and disorders. In a recent study, Banerjee et al. showed that MSC-Exo treatment increased IL-10 secretion and M2-polarized macrophages in the hearts of mice with diabetic cardiomyopathy. The treated mice exhibited reduced TAK1-pJNK-NFKB inflammation-associated expression and improved cardiac function with significantly reduced cardiac hypertrophy and fibrosis compared to non-treated diabetic mice [104]. Sun et al. also found that MSC-Exo improved the inflammatory microenvironment of doxorubicin-induced dilated cardiomyopathy by regulating the polarization of the macrophage [105].

### 2.3. MSC-Exo Promote Angiogenesis in Myocardial Injury

#### 2.3.1. MSC-Exo in Angiogenesis

Angiogenesis is the formation of new blood vessels on the basis of existing vasculature. This process involves several steps, such as the activation, migration, and proliferation of vascular endothelial cells. Angiogenesis is crucial for repairing cardiac injuries and improving cardiac function. Many studies have demonstrated that exosomes from MSCs promote angiogenesis in MI and other cardiac injuries. Teng et al. showed that intramyocardial injection of MSC-Exo promoted the density of new functional capillaries, reduced infarct size, and preserved cardiac systolic and diastolic function in a rat MI model [84]. Similarly, in their study, Ju et al. demonstrated that MSC-Exo preserved heart function in acute MI mice by enhancing cardiac angiogenesis and cardiomyocyte proliferation [107]. Mechanistically, different signaling effector molecules have been reported in different studies. In a recent study, miR-205 signaling was implicated in the proliferation and migration of microvascular endothelial cells to facilitate angiogenesis. The study showed that exosomes from adipose-derived mesenchymal stem cells promoted angiogenesis and alleviated myocardial injury in mice with MI via miR-205 signaling [108]. Upregulation of angiogenic factors and their signaling play an important role in MSC-Exo-mediated angiogenesis. Hu et al. found that MSC-Exo has proangiogenic effects on endothelial cells. Their study revealed exosome treatment upregulated the expression of angiogenic factors HIF-1α and VEGF in the left ventricle and enhanced angiogenesis in rats with heart fibrosis induced by isoproterenol (ISO) [109]. The angiogenic ability of MSC-Exo might be dose-dependent. In a study by Bian et al., MSC-EV were promptly uptaken by endothelial cells, and the internalization resulted in dose-dependent enhancement of proliferation, migration, and tube formation of endothelial cells in vitro. The authors also showed, in line with other studies, that intramyocardial injection of MSC-EV enhanced blood vessel formation and preserved cardiac function in MI-induced injury in rats [110].

#### 2.3.2. MicroRNAs as Critical Mediators in MSC-Exo-Induced Angiogenesis and Cardiac Repair

Numerous studies demonstrated that microRNAs are the key messengers in the MSC-Exo/EV in modulating angiogenesis via regulating different angiogenesis-related signaling pathways. Yang et al. showed that MSC-EV facilitated angiogenesis in cardiac microvascular endothelial cells (CMEC) in ischemic injury via transfer of the specific microRNA miR-543. Their study demonstrated that intravenous injection of MSC-EV promoted cardiac repair in rats with MI by transferring miR-543 into myocardial tissues or cardiac microvascular endothelial cells and downregulating the expression of COL4A1, a protein associated with ischemia [111]. Another study reported a decrease in miR-29b-3p expression in the myocardial tissues in MI rats; however, MSC-Exo’s upregulation of miR-29b-3p improved myocardial angiogenesis and ventricular remodeling by regulating ADAMTS16, a negative regulator of fibrosis and cardiac function [112].

The presence of certain microRNAs as a part of the natural cargo of MSC-Exo proved vital in promoting angiogenesis. Wang et al. studied the effects of MSC-Exo on a chronic heart failure mouse model and identified the critical role of miR-1246 in cardiac repair. The study showed that exosomes derived from human umbilical cord mesenchymal stem cells (hucMSC-Exo) promoted angiogenesis and inhibited myocardial injury in chronic heart failure (CHF) mice. HucMSC blocked Snail/α-SMA signaling by delivering miR-1246 to enhance angiogenesis and protect cardiomyocytes, whereas silencing miR-1246 in hucMSC inhibited the beneficial effects of exosomes [113]. Another similar study revealed that upregulation of miR-486-5P in small extracellular vesicles (sEV) derived from hypoxia-preconditioned MSC enhanced angiogenesis and improved cardiac function in rodent and nonhuman primate models of MI. Conversely, the inactivation of miR-486-5p abolished the benefits [114]. Consistently, silencing of other microRNAs, such as miR-210-5p and miR-210-3p, also resulted in the impairment of MSC-Exo’s angiogenesis ability during cardiac repair, confirming the critical role of specific microRNAs [43,115,116].

Interestingly, human bone marrow MSC-derived exosomes from young donors were more effective in in vitro endothelial tube formation and improvement of cardiac function in the MI rats compared to exosomes from aged donors [117]. This was linked to miR-221-3p being downregulated in aged exosomes. Strikingly, the cardiac reparative ability of aged exosomes was restored when miR-221-3p was upregulated [117].

## 3. Approaches to Enhance the Cardioprotective Effects of MSC-Exo

### 3.1. Genetic Modification

#### 3.1.1. Upregulation of Gene Expression

**Figure 2:**
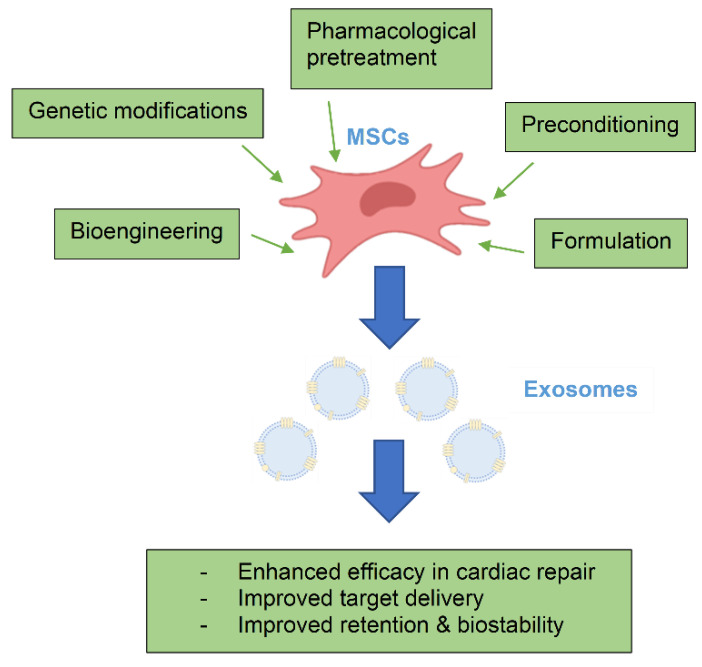
Approaches to enhance the cardioprotective effects of MSC-Exo.

Upregulation of gene expression has been one of the most studied approaches to enhance the therapeutic effects of MSC-Exo (Table 3). This involves overexpression of genes in the MSCs that positively impact cardiac repair through various pharmacological effects, including cellular regulation, angiogenesis, inflammation, and cytoprotection. Studies have demonstrated that exosomes derived from these modified MSCs exhibit superior cardioprotective action in myocardial injury compared to exosomes from non-modified MSCs (Figure 2).

HIF-1α is a key transcriptional regulator for gene expressions in response to hypoxia. It regulates several gene expressions, including those encoding angiogenesis and cell proliferation. Sun et al. studied the effects of HIF-1α modification on MSC-Exo and showed that HIF-1α overexpression in MSC led to better preservation of heart function by exosomes compared to exosomes from non-modified MSC in a rat MI model. Modified exosomes showed enhanced promotion of angiogenesis and inhibition of fibrosis [118]. Macrophage migration inhibitory factor (MIF) promotes cell proliferation and survival. Two studies demonstrated that MSC-Exo with overexpression of MIF had better cardiac protection in rats with AMI [119,120].

Other research also confirmed that upregulating certain genes in MSCs led to better cardiac repair through exosomes. These studies highlighted the involvement of different mechanisms in providing cardioprotective effects. In MI mice, overexpressing FNDC5, a transmembrane protein located in the cytoplasm, enhanced MSC-Exo’s capacity to polarize M2 macrophages compared to non-modified MSC-Exo [98]. After MI, injured cardiomyocytes express stromal-derived factor 1 (SDF1), a stem cell chemical attraction factor, to aid in cardiac repair. SDF1 can engage and mobilize CXCR4-expressing bone marrow stem cells and thereby promote revascularization in ischemic injury [15,121]. Exosomes, derived from either SDF-1 or CXCR4 overexpressing MSCs, were able to provide better cardioprotection than non-modified MSC-Exo in rodent MI, and the same PI3K/Akt signaling pathway was involved in conferring the beneficial effects, as shown in two separate studies [122,123]. Tissue matrix metalloproteinase inhibitor 2, also known as TIMP2, is a key determinant of post-MI remodeling. Exosomes derived from TIMP2-overexpressing human umbilical cord MSC significantly improved cardiac function in the MI rat by promoting angiogenesis and inhibiting extracellular matrix (ECM) remodeling, partly via the Akt/Sfrp2 pathway [124]. Consistent with these studies, upregulation of other genes, such as GATA-4, Nrf2, and Akt, in the MSCs has been shown to provide better cardioprotection and improved cardiac function recovery in different myocardial injuries [45,125,126].

#### 3.1.2. microRNA/Non-Coding RNA Regulation

MicroRNAs (miRNAs) have been proven to be effective therapeutic agents for MI and other cardiac injuries (Table 4). For instance, accumulating evidence suggests that miR-146a protects the myocardium in different cardiac injuries. Wang et al. showed that miR-146a transfection in hearts improved cardiac function in mice with I/R injury by attenuating apoptosis and inhibiting inflammatory cytokine production through suppression of IRAK1 and TRAF6 and inhibition of NF-κB activation [127]. Consistently, another study demonstrated that miR-146a-containing milk exosomes improved cardiac function in a rat model of myocardial I/R injury by inhibiting the IRAK1/TRAF6/NF-κB signaling pathway [128]. Studies have also reported the protective role of miR-146a in drug-induced cardiotoxicity. Pan et al. showed that overexpression of miR-146a inhibited doxorubicin (DOX)-induced cardiotoxicity, while the injury was more severe when miR-146a was downgraded [129]. MiR-146a-deficient mice showed worse cardiac function compared to wild-type mice in the DOX-induced injury model. Mechanistically, miR-146a partially reversed the DOX-induced cardiotoxicity by targeting the TAF9b/P53 pathway to attenuate apoptosis and adjust autophagy levels [129]. In another study, miR-146a attenuated isoproterenol-induced cardiac fibrosis by inhibiting FGF2 [130]. A study on transgenic mice with specific overexpression of TNF-α in the heart showed increased expression of miR-146a. miR-146a directly targeted Fos to inhibit MMP-9 activity, which is involved in cardiac remodeling, myocardial dysfunction, and progression of heart failure. Thus, the study suggested a protective role of miR-146a in cardiac disorders associated with enhanced inflammation in the heart [131]. Studies with sepsis-induced injury also confirmed the cardioprotective role of miR-146a. Liu et al. showed that miR-146a attenuated cardiomyocyte apoptosis and inflammatory response in LPS-induced sepsis injury via decreasing MYBL1 expression [132]. In another study, miR-146a deficiency promoted aged neutrophil phenotype and thrombosis in atherosclerosis and LPS-induced inflammatory injury mouse models, suggesting its critical role in the regulation of inflammation in adverse cardiovascular events [133].

However, the poor stability of miRNAs in the body and limited cellular uptake hinder their clinical application [134,135]. Exosomes, which can deliver specific miRNAs to recipient cells, offer advantages as cell-free delivery tools. One of the benefits of exosome-mediated miRNA delivery is the lower risk of triggering inflammatory immune responses and other unwanted side effects, such as carrier toxicity, commonly associated with viral and non-viral-based miRNA delivery. Moreover, exosomes demonstrate good biocompatibility, safety, and stability, making them an ideal vehicle for delivering miRNAs [134,136,137]. A number of studies have shown that MSC-Exo mediates cardioprotection in myocardial injury via delivering miRNAs. Therefore, studies have also focused on upregulating specific miRNAs in the MSCs to deliver those specific miRNAs through the secreted exosomes in myocardial injury.

Upregulated miR-146a in the MSC-Exo from miR-146a-modified MSCs resulted in improved cardioprotection in rat MI injury [138]. MiR-214 plays an important role in mediating anti-apoptosis and angiogenesis. Zhu et al. showed that miR-214 overexpression in MSCs enhanced the therapeutic efficacy of MSC-Exo in AMI by promoting cardiomyocyte survival and endothelial cell function via targeting PTEN and activating the p-AKT signaling pathway [139]. Similarly, exosomes derived from miR-129-5p-overexpressing MSCs have been shown to improve cardiac function, reduce inflammation, and provide better cardiomyocyte protection in MI mice by decreasing the expression of HMGB1 [140]. In myocardial injury, the expression of some microRNAs goes down. Upregulation of the expression of these microRNAs in MSCs has been shown to confer better cardioprotective effects by MSC-Exo. MSC-Exo can deliver these microRNAs to the site of injury and help restore their expression. For example, miR-19a is downregulated in AMI tissues and cells. MSC-Exo can transfer miR-19a to cardiomyocytes, protecting them from MI injury [141]. Additionally, MSC-Exo with low expression of miR-19a showed decreased myocardial protection in AMI rats, while MSC-Exo with overexpression of miR-19a enhanced the protection. This further demonstrated that the upregulation of miR-19a in the MSCs could provide better cardiac repair effects [141]. Exosomes secreted by bone marrow MSCs transfected with miR-301 mimics were shown to increase miR-301 expression in MI tissues of rats. These exosomes showed enhanced protection in MI compared to unmodified MSC-Exo by inhibiting autophagy [40]. Consistently, other studies involving the upregulation of different microRNAs, such as miR-200b-3p and miR-19a/19b, also showed enhanced cardioprotection in MI injury [93,142].

Although transfection has been the most common approach to upregulate specific miRNAs in the MSC-Exo, electroporation of miRNA directly into the exosomes has also been effective. Ma et al. found that miR-132 can be effectively incorporated into MSC-Exo by electroporation and can be transferred to the recipient cells [143]. MiR-132-loaded MSC-Exo showed increased angiogenesis capacity in vivo, and their transplantation in the ischemic hearts of mice resulted in enhanced neovascularization in the peri-infarct zone and preserved heart functions in a mouse MI model [143].

Upregulation of long non-coding RNA in the MSC-Exo has also been studied. For instance, Mao et al. showed that intravenous injection of exosomes from lncRNA KLF3-AS1-overexpressing MSC in MI rats led to reduced MI area, decreased cell apoptosis and pyroptosis, and attenuated MI progression. Interestingly, KLF3-AS1 could sponge miR-138-5p to regulate Sirt1 expression to attenuate pyroptosis and MI progression, suggesting that miR-138-5p is a negative modulator and can be a target in MI injury [99].

### 3.2. Preconditioning

Modified exosomes from preconditioned MSCs, including pharmacological pretreatment and hypoxic culture, have been shown to confer additional benefits compared to non-modified exosome treatment in various preclinical models of cardiovascular disease. Studies have shown that exosomes derived from pre-treated MSCs can attenuate myocardial injury and improve cardiac function in MI and ischemia-reperfusion injury [144,145].

A study with exosomes derived from atorvastatin (ATV)-pretreated MSCs (ATV-MSC) enhanced the migration and tube formation of endothelial cells and increased their survival. The authors demonstrated that ATV-pretreated MSC-Exo improved recovery in cardiac function and reduced infarct size in MI-induced heart injury compared to exosomes from regular MSCs [146]. Consistently, a study by Ning et al. showed that ATV-pretreated exosomes improved post-MI left ventricle ejection fraction and fractional shortening better than exosomes from non-modified MSCs [96]. Similarly, pre-treatment with other pharmacological agents, including empagliflozin, hemin, irisin, IFN-γ, tongxinluo, and tanshinone IIA, has been studied in various myocardial injuries, showing consistent beneficial results in cardiac repair [147,148,149,150,151,152]. The enhancement of efficacy by preconditioning is mediated via different mechanisms and is associated with several beneficial outcomes, including enhanced angiogenesis, reduction of inflammation, inhibition of apoptosis, and promotion of cell survival. ATV-treated MSC-Exo promoted endothelial cell function, and this is associated with increased VEGF levels [153]. Exosomes derived from ATV-pretreated and lipopolysaccharide (LPS)-pretreated MSC had better macrophage M2 polarization compared to non-modified MSC-Exo [96,97]. The expression of inflammation markers such as TNF-α and IL-6 was more effectively suppressed by ATV-treated MSC-Exo as well [97]. Upregulation of certain therapeutic miRNAs and long non-coding RNAs (lncRNAs) within modified exosomes has been highlighted in mediating superior cardioprotection. For example, the elevated level of lncRNA H19 in the ATV-treated exosomes played a key role in promoting angiogenesis [146]. Similarly, elevated exosomal miR-214-3p from empagliflozin-pretreated MSCs mediated, at least partially, the cardioprotective effects by inhibiting apoptosis [147].

Similar to pharmacologic interventions, hypoxia preconditioning has also been shown to induce changes in the composition and cargo of exosomes, potentially enhancing their cardioprotective effects. It is well documented that hypoxia can upregulate the expression and release of paracrine factors within MSC-Exo that provide cardioprotective actions and enhance myocardial repair [42,44,154,155]. For example, a study by Zhu et al. showed that exosomes from hypoxia-conditioned MSCs enhanced cardioprotective functions in MI rats. They found that hypoxia-conditioned MSC-Exo contained enriched miR-125b-5p, which played a crucial role in anti-apoptosis and cardioprotection [42]. Another study also confirmed that hypoxic preconditioning exerted decreased apoptosis and improved cardiac functions in rat MI injury [44]. Two separate studies by different groups showed that hypoxic culture upregulated miR-210 in MSCs and their secreted exosomes compared to normoxic culture, and miR-210 was responsible at least in part for the enhanced cardioprotective effects [43,154].

### 3.3. Formulation/Delivery

The short half-life of exosomes, low retention in desired tissue sites, and rapid clearance in vivo remain major challenges in clinical applications. For instance, direct intramyocardial injections have the risk of rapid wash-out from the myocardium, potentially weakening their therapeutic efficacy. Similarly, intravenous administration might not achieve targeted effects and could lead to accumulation in other organs [155,156]. Determining optimal exosome dosage could also be challenging for clinical application. To address these challenges, different formulation and delivery strategies have been studied, such as incorporating exosomes into bioactive hydrogel or engineering them with biocompatible compounds for cell/tissue-specific targeted delivery.

A study by Zou et al. showed that formulating MSC-Exo into an injectable conductive hydrogel could prolong their retention when injected into the injured hearts of rats [157]. The exosome-loaded hydrogel system improved cardiac functions by promoting cell proliferation and angiogenesis in MI-I/R injury. The hydrogel provided conductivity matching the native myocardium, stability adapting to heartbeats, and better cytocompatibility as well [157]. Other studies have also reported enhanced efficacy with hydrogel formulation. MSC-Exo loaded in different hydrogels, such as hyaluronic acid, angiogenin-1, and alginate, could boost the retention of exosomes and further enhance their therapeutic effects in MI injury compared to intramyocardial administration of non-modified exosomes [158,159,160].

Exosome-loaded hydrogels are usually administered directly into the infarcted myocardium to ensure that the exosomes are applied locally and reliably at the site of injury. However, intramyocardial injections are associated with the risk of secondary injury. Considering this, Zhu et al. studied the impact of intrapericardial delivery. The study showed that intrapericardial injection of biocompatible hydrogel containing MSC-Exo can form a cardiac patch-like structure in the pericardial cavity and increase the cardiac retention of exosomes in rodents. The authors suggested that the pericardial cavity acted as a natural mold, and the injection formed a structure in situ, matching the shape of the heart [161]. This delivery method improved cardiac functions post-MI. The study also demonstrated the feasibility and safety of this formulation in a clinically relevant porcine model [161]. In a separate study, the same group demonstrated that this formulation also reduced LV chamber size and preserved wall thickness in aged rats with TAC-induced heart failure [162].

Implantation of MSC-EV-loaded 3D scaffolds engineered with decellularized cardiac tissue could locally deliver EVs to the infarcted myocardium of pigs. The integration of the implant into the post-infarcted myocardium generated a bioactive niche, promoted vascularization, recruited pro-regenerative cells, and reduced inflammatory responses [163]. Another study found that implanting a gelatin-based microneedle patch containing MSC-Exo loaded with miR-29b mimics could increase the retention of loaded exosomes in the infarcted myocardium, thereby improving cardiac function [164].

Wang et al. fused an ischemic myocardium-targeting peptide, CSTSMLKAC (IMTP), with exosomal membrane protein Lamp2b and introduced the sequence in the MSCs to guide the exosomes into ischemic cardiac cells. Their study showed that exosomes derived from the engineered MSCs could be more efficiently internalized by ischemic cardiomyocytes and thereby enhance therapeutic effects in acute MI mice [165]. In a recent study, Gu et al. showed that miR-302-loaded MSC-Exo, engineered with cardiomyocyte-specific peptide by mixing in an ethanolic solution of phospholipid, could be more efficiently internalized by cardiomyocytes compared to unmodified exosomes. The modified exosomes provided improved therapeutic function in mouse MI [166]. Exosomes with overexpression of the membrane protein CD47 can promote immune evasion, and they have an extended circulation half-life in mice [167,168]. Wei et al. studied the biodistribution and delivery efficiency of MSC-EV-carrying CD47 and anti-apoptotic miRNA miR21. Their study showed that EVs derived from MSCs overexpressing CD47 (CD47-EVs) were still detectable in the plasma 120 min after the tail vein injection, compared to the detection time of less than 30 min with the unmodified EVs. Intravenous injection of miR21-loaded CD47-EVs preferentially accumulated in the heart in the ischemia-reperfusion region, resulting in significant anti-apoptotic effects and reduced cardiac inflammation with improved cardiac function [103].

Finally, the yield of MSC-Exo/EV is a limiting factor for large-scale production in clinical applications. To address this, Sun et al. showed that hollow fiber bioreactor-based 3D culture of human umbilical cord-derived MSC yielded higher EVs compared to 2D culture. Three-dimensional-EVs showed similar biofunctions compared to 2D-EVs and were able to protect cardiomyocytes and improve cardiac function in AMI rats [169].

## 4. Limitations/Challenges in Clinical Application

Although many preclinical studies have demonstrated that MSC-Exo can provide cardioprotection in different cardiac injuries, a few issues need to be addressed before their successful implementation in clinical settings.

There are several techniques to isolate exosomes, such as ultracentrifugation, size exclusion chromatography, ultrafiltration, and precipitation, but there is no unified standard. Ultracentrifugation is low-cost and the most common method for isolating exosomes, but it has several limitations. The yield is low compared to other methods, such as size-exclusion chromatography and ultrafiltration. The process has multiple steps and thus is labor-intensive and time-consuming [170]. Similarly, other techniques have their own advantages and disadvantages. Heterogeneity and purity are other major technical challenges in isolating a pure exosome population. Since most methods separate exosomes based on their size and density, the isolated exosomes may contain contamination from particles of similar size and density, such as microvesicles, small apoptotic bodies, and non-exosomal proteins. Additionally, cell culture conditions such as passage and media, different isolation methods, and the type of equipment used can result in both quantitative and qualitative variation in yield. Overall, heterogeneity, contaminations, low yield, and lack of standardization in isolating exosomes are important drawbacks of using MSC-Exo in clinical settings [170,171,172].

Delivering MSC-Exo to target sites and maintaining its efficacy remains a major challenge. Studies have estimated the half-life of exosomes is as low as 2–30 min, and 90% of exosomes might be removed within 5 min after infusion [173,174,175,176,177]. Due to the inherent short half-life of exosomes and since they are quickly removed from circulation, it is challenging to deliver them to injury sites as well as maintain the therapeutic level. Thus, the frequency of dosing to maintain the therapeutic level in the body needs to be standardized. To address this issue, different delivery methods have also been studied with success in preclinical settings, such as formulating MSC-Exo into biocompatible hydrogel, loading them into scaffolds or microneedle patches, and fabricating or engineering them to guide into specific tissue sites (Section 3.3). However, these delivery strategies need to be standardized, and more studies are needed before implementing them into clinical practices.

The lack of scalability is another major challenge that is hindering the clinical translation of MSC-Exo. As shown in Table 1 and Table 2, most of the preclinical studies with MSC-Exo are performed with microgram amounts, but this is not sufficient for clinical settings. To upscale the production of exosomes from cells, various approaches have been studied, including hollow-fiber bioreactors and other 3D cultures, chemical and physical fabrication, preconditioning, and genetic manipulation of source cells. However, this scale-up process might alter the MSC phenotype and the downstream biological function of exosomes. For example, there are reports that the shear stress in a bioreactor can modify the MSC phenotype [178,179]. A key technical challenge for this up-scaling is the need to control the environmental parameters so that the yields are of high quality and without significant impurities. Good manufacturing practices must be ensured to address these issues before using the MSC-Exo in clinical settings.

Different tissue sources have been used for the functional analysis of MSC-Exo in cardiac repair in different studies; however, cargos can be different depending on the source and therefore might provide repair effects to different degrees [180,181]. For example, Wang et al. reported that exosomes from human endometrium-derived MSC confer better cardioprotection than exosomes from adipose or bone marrow-derived MSCs [182]. Therefore, more comparative studies on the therapeutic effects of different tissue sources are warranted, and standardization of the source based on the functionality might be beneficial in the context of clinical application. Also, establishing the best route of delivery, optimal dose, and therapeutic window is necessary. Table 1 and Table 2 illustrate that various studies have utilized varying doses, potentially due to variations in sources, extraction methods, and usage routes. Therefore, it is crucial to establish a standard dose for clinical applications.

The comparison between MSC-Exo and pharmacological treatments in cardiac repair is meaningful but remains underexplored. Pharmacological treatments, such as sodium-glucose cotransporter 2 (SGLT2) inhibitors, have demonstrated significant cardioprotective effects in various preclinical models of myocardial injury. Notably, the SGLT2 inhibitor empagliflozin was shown to reduce infarct size (33% in control vs. 17% in the treatment group) and improve cardiac outcomes in mice with I/R injury, as reported by Andreadou et al. in 2017 [183]. Moreover, empagliflozin received FDA approval for heart failure treatment in 2021, signaling its clinical relevance and effectiveness in heart disease management. On the other hand, MSC-Exo has also shown promise in repairing myocardial damage. These exosomes have been observed to improve cardiac function, enhance ejection fraction, reduce infarct size, and protect cardiomyocytes in both acute and chronic injury models. Numerous studies referenced in our manuscript highlight these benefits across various preclinical models. Future research is essential to compare the efficacy of MSC-Exo and pharmacological treatments in cardiac repair.

Most research has focused on examining how the exosomal content modulates signaling pathways associated with cell death, angiogenesis, and inflammation; however, the precise molecular mechanisms, particularly those upstream of these signaling cascades, remain understudied. Currently, unlike single-cell analysis, there is no suitable method for single-exosome analysis. Studying cargos from a single exosome would provide a better understanding of the heterogeneity in the whole exosome population and might shed light on the molecular mechanism.

Therefore, further research is needed to optimize the isolation and usage strategies and gain a comprehensive understanding of the mechanisms underlying the cardioprotective effects of MSC-Exo to translate the preclinical research findings into clinical applications for the treatment of myocardial injury.

## 5. Conclusions

MSC-Exo, as one of the key paracrine effectors, has been a major focus of MSC-mediated therapeutic effects in recent years. There is plenty of preclinical evidence showing the efficacy of MSC-derived exosomes in animal models of myocardial injury. They exert multiple therapeutic benefits, including protection against cardiomyocyte death, anti-inflammation, and angiogenesis to mitigate injury. Their cargos, including proteins and especially miRNAs, are of interest since many studies have shown that MSC-Exo can deliver them to target sites to regulate important protein expression and cellular signaling. Manipulation of these cargos, such as upregulation of specific genes or miRNAs, has resulted in better efficacy, as shown in numerous studies. However, studies have primarily focused on the modification of a single gene or miRNA of interest in the MSC-Exo. Therefore, it would be interesting to see the outcome associated with multiple gene editions simultaneously targeting a specific cardiac repair pathway using gene-editing tools like CRISPR-Cas9. Different formulation approaches, such as preconditioning, and exosome engineering to enhance retention and site-specific delivery, also showed better cardioprotective effects. Although the preclinical studies are promising, there is still a broad gap between experiments and the clinical application of MSC-Exo in myocardial injury. Standardization of the tissue source, isolation method, dosage, and delivery routes, and understanding the precise molecular mechanism will help close this gap. Currently, there is only one ongoing clinical trial (NCT05669144) evaluating the effects of MSC-Exo in MI. Therefore, it is necessary to design and conduct more clinical studies to ensure the safety and efficacy of MSC-Exo therapy in different cardiac injuries in humans.

In summary, the unique properties of MSC-Exo, including their ability to reach any tissue to deliver their specific cargo and the promising preclinical results, have made the prospect of this therapy for myocardial injury exciting. Although the transition from preclinical studies to clinical practice in patients requires more challenges to overcome, the future holds promise.

## Figures and Tables

**Table 1:** MSC-Exo provides cardiomyocyte protection following myocardial injury.

Model of Myocardial Injury	MSC-Exo/EV Source	Dose	Administration	Mechanisms/Signaling Pathway	Related miRNA	Effects	Author
Myocardial Infarction (MI)							
Rats MI by LAD ligation	Human bone marrow	5 × 10^10^ EV particles, 4 doses	Intraperitoneal	Inhibit ZFAS1 and activate Akt/Nrf2/HO-1 pathway		Inhibit apoptosis, and improve cell migration and proliferation; improve morphological damage observed in MI	Xiao et al. (2022) [33]
Mice MI by LAD ligation	Mouse bone marrow	Exosomes from 1 × 10^6^ MSCs, 2 doses	Intramyocardial Immediately after LAD ligation + tail vein injection after 24 h	Activate macrophage phagocytosis ability through Milk Fat Globulin Epidermal Growth Factor VIII (MFGE-8)/Integrin signaling		Opsonize dead cardiac cells and enhance their engulfment and removal by immune cells. Enhance inflammation resolution.	Patil et al. (2021) [34]
Mice MI by LAD ligation	Mouse bone marrow	20 μg	Tail vein injection after 48 h of MI induction	Alter expression of miRNAs associated with cell differentiation and apoptosis		Decrease apoptosis and improve myocardial function	He et al. (2018) [35]
Rats MI by LAD ligation	Human umbilical cord	400 μg following MI	Intramyocardial	Promote fibroblast-to-myofibroblast differentiation		Attenuate inflammatory responses and decrease apoptosis	Shi et al. (2019) [36]
Rats MI + Cecal ligation and puncture (CLP)				Upregulate circRTN4/miR-497-5p/ MG53 pathway	miR-497-5p	Suppress upregulated reactive oxygen species (ROS) and apoptosis	Li et al. (2022) [37]
Mice MI by LAD ligation		5 μg, 30 min after ligation	Intramyocardial	Modulate p53-Bnip3 signaling	miR-125b	Improve autophagic flux and cardiac function	Xiao et al. (2018) [38]
Rats MI by LAD ligation	Adipose tissue	400 μg following MI	Intravenous	Suppress Atg7 and TLR4/NF-κB pathway	miR-93-5p	Inhibit autophagy and inflammatory responses	Liu et al. (2018) [39]
Rats MI by LAD ligation	Rat bone marrow		Intramyocardial		miR-301	Inhibit autophagy and improve cardiac function	Li et al. (2019) [40]
Mice MI by LAD ligation	Human umbilical cord	5 μg	Intramyocardial	Suppress divalent metal transporter 1 (DMT1) expression	miR-23a-3p	Inhibit ferroptosis and attenuate myocardial injury	Song et al. (2021) [41]
Mice MI by LAD ligation	Mouse bone marrow	200 μg/ 20 g body wt.	Intramyocardial	suppress the expression of the proapoptotic genes p53 and BAK1 in cardiomyocytes	miR-125b-5p	Ameliorate cardiomyocyte apoptosis and facilitate ischemic cardiac repair	Zhu et al. (2018) [42]
Rats MI by LAD ligation	Rat bone marrow	Exosomes from 10^6^ MSCs	Cardiac	Downregulate levels of p-AKT, p-PI3K, and p-p53 by downregulating AIFM3	miR-210	Inhibit apoptosis and improve cardiac function	Cheng et al. (2020) [43]
Rats MI by LAD ligation	bone marrow		Intramyocardial	Downregulate expression of apoptotic markers Bax, Caspase-3, and cleaved-Caspase-3	miR-24	Inhibit apoptosis and improve cardiac function	Zhang et al. (2019) [44]
Rats MI by LAD ligation	Rat bone marrow	Exosomes from 4 × 10^6^ MSCs	Intramyocardial	Suppress expression of PTEN; activate Akt and ERK signaling pathways	miR-19a	Reduce apoptosis and promote cardiac function recovery	Yu et al. (2015) [45]
Ischemia- Reperfusion (I/R) injury							
Rats I/R injury by LAD ligation	Rat adipose tissue	400 μg within 5 min of reperfusion	Intravenous (Tail vein)	Activate Wnt/β-catenin signaling pathway		Inhibit apoptosis	Cui et al. (2017) [46]
Rats I/R injury by LAD ligation	Rat bone marrow	10 μg	Intramyocardial	Suppress MEKK1-MKK4-JNK Signaling pathway	miR-455-3p	Suppress apoptosis and autophagy	Wang et al. (2022) [47]
Rats I/R injury by LAD ligation	Rat bone marrow	200 μg	Intramyocardial	Suppress CHK2-Beclin2 pathway	miR-143-3p	Inhibit autophagy and reduce myocardial I/R injury	Chen et al. (2021) [48]
Rats I/R injury by LAD ligation	Rat bone marrow	5 μg	Intramyocardial	Modulate AMPK/mTOR and Akt/mTOR signaling		Induce autophagy, decrease apoptosis and improve heart function	Liu et al. (2017) [49]
Rats I/R injury by LAD ligation	Human bone marrow	500 μg 5 min before reperfusion	Intramyocardial	Disinhibit IGF1/PI3K/AKT pathway	miR-497	Reduce apoptosis and suppress myocardial H/R injury	Li et al. (2021) [50]
Rats I/R injury by LAD ligation		Exosomes from 5.8 × 10^12^ particles, 10 min before perfusion	Intramyocardial	Downregulate expression of thioredoxin-interacting protein (TXNIP)	miR-150-5p	Inhibit cardiomyocyte apoptosis and myocardial remodeling following I/R injury	Ou et al. (2020) [51]
Rats I/R injury by LAD ligation	Rat bone marrow	400 μg within 5 min of reperfusion	Intravenous (Tail vein)	Inhibit FOXO1 expression	miR-183-5p	Reduce apoptosis and oxidative stress in I/R cardiomyocytes and improve cardiac function	Mao et al. (2022) [52]
Rats I/R injury by LAD ligation	Rat bone marrow	400 μg at the start of reperfusion	Intravenous (Tail Vein)	Suppress PTEN expression and activate PI3K/AKT pathway	miR-486-5p	Inhibit cardiomyocyte apoptosis	Sun et al. (2019) [53]
Miscellaneous In vivo injury model							
Transverse aortic ligation-induced pressure overload in mice	Mouse bone marrow		Intramyocardial			Attenuate apoptosis and cardiac hypertrophy	Chen et al. (2020) [54]
Cecal ligation and puncture (CLP) induced sepsis cardiomyopathy	Human umbilical cord	2 μg/g body wt. at 0 h and 6 h after CLP		Transfer PINK1 mRNA to cardiomyocytes to increase its expression; provide mitochondrial calcium homeostasis		Prevent cardiomyocyte mitochondrial damage and protect the heart from sepsis-induced injury	Zhou et al. (2021) [55]
Coxsackievirus B3 (CVB3)-induced myocarditis	Human umbilical cord	50 μg	Intravenous	Activate AMPK/mTOR-mediated autophagy flux pathway		Attenuate cardiomyocyte apoptosis and decrease inflammatory response	Gu et al. (2020) [56]
Cyclophosphamide (CYP) induced cardiotoxicity in rats	Adipose tissue	2 doses; from 3 × 10^10^ MSC, one before CYP inj., and one after				Repress oxidative stress, apoptosis, and autophagy; improve cardiac function	Xiong et al. (2023) [57]
In vitro injury model							
High-frequency electrical stimulation to induce atrial fibrillation in vitro	Mouse bone marrow			Inhibit expression of SPARC- associated modular calcium-binding protein 2 (SMOC2)	miR-148a	Inhibit cardiomyocyte apoptosis	Zhang et al. (2022) [58]
In vitro hypoxia	Rat bone marrow			Apoptosis signal-regulated kinase-1 (ASK1) ubiquitination		Inhibit cardiomyoblast apoptosis and promote viability	Li et al. (2023) [59]
In vitro hypoxia- regeneration (H/R) injury	Human umbilical cord	8 μg/mL before H/R injury induction		Activate PI3K/Akt signaling pathway		Inhibit endoplasmic reticulum (ER) stress and apoptosis	Zhang et al. (2020) [60]
In vitro study of Doxorubicin induced cardiotoxicity	Human umbilical cord	50/100/200 μg/mL		Activate miR-100-5p/NOX4 pathway	miR-100-5p	Suppress oxidative stress and apoptosis	Zhong et al. (2021) [61]
In vitro cardiomyocyte I/H injury	Bone marrow			Modulate ROCK1 expression and stimulate the PI3K/AKT/mTOR Signaling pathway	miR-144-3p	Inhibit I/H-induced cardiomyocyte apoptosis and autophagy	Wang et al. (2023) [62]
In vitro hypoxia- regeneration injury	Rat bone marrow			Inhibit Faslg and promote w/β-catenin signaling pathway	miR-149/let-7c	Protect cardiomyocyte against H/R induced ROS production and apoptosis	Zou et al. (2020) [63]
In vitro hypoxia- regeneration injury				Increase XIAP expression and regulate miR-556-5p/XIAP	miR-556-5p	Attenuate H/R-induced apoptosis and oxidative stress	Yu et al. (2023) [64]
In vitro hypoxia- regeneration injury	Bone marrow			Activate HAND2-AS1/miR-17-5p/Mfn2 pathway	miR-17-5p	Reduce apoptosis, oxidative stress, and represses inflammation	Li et al. (2023) [65]

**Table 3:** Gene upregulation to enhance the efficacy of MSC-Exo in cardiac repair.

Upregulated Gene	Model of Myocardial Injury	MSC-Exo/EV Source	Administration	Related miRNA	Mechanisms/Signaling Pathway	Effects
HIF-1α	Rats MI by LAD ligation	Rat bone marrow	Intravenous (Tail-vein)		VEGF/PDGF	Promote angiogenesis and inhibit fibrosis
MIF	Rats MI by LAD ligation	Human umbilical cord	Intramyocardial	miR-133a-3p	VEGF, Akt Signaling	Promote angiogenesis, inhibit apoptosis, reduce fibrosis, and preserve heart function
MIF	Rats MI by LAD ligation	Human bone marrow	Intramuscular		Activate AMPK signaling, Inhibit mitochondrial fission	Inhibit apoptosis
FNDC5	Mice MI by LAD ligation	Mouse bone marrow	Intramyocardial		Suppress the NF-κB signaling pathway and upregulate Nrf2/HO-1 signaling; increase M2 macrophage polarization	Attenuate apoptosis, inflammation, and improve cardiac function
SDF	Mice MI by LAD ligation	Human umbilical cord	Intramyocardial		Activate PI3K signaling	Inhibit apoptosis and autophagy and preserve cardiac function
TIMP2	Rats MI by LAD ligation	Human umbilical cord	Intramyocardial		Downregulate MMP2 and MMP9 expression, activate Akt/Sfrp2 pathway	Attenuate apoptosis, alleviate cardiac remodeling, and dysfunction
GATA-4	Rats MI by LAD ligation	Rat bone marrow	Intramyocardial	miR-19a	Downregulate PTEN and BIM expression	Reduce apoptosis, promote angiogenesis, and cardiac function recovery
CXCR4	Rats MI by LAD ligation	Rat bone marrow	Exosome pretreated cell patch graft		Activate PI3K/Akt signaling pathway	Inhibit apoptosis, promote angiogenesis, and reduces infarct size
Akt	Rats MI by LAD ligation	Human umbilical cord	Intravenous (Tail-vein)		Promote PDGF expression	Promote angiogenesis and improve cardiac function
Nrf2	Rats atrial fibrillation (AF) by injection of calcium chloride- acetylcholine	Rat bone marrow	Intramyocardial		Modulate the Nrf2-HO-1 pathway	Inhibit arrhythmias in AF; inhibit apoptosis, inflammation, and fibrosis in AF rats

**Table 4:** MicroRNA regulation to enhance the efficacy of MSC-Exo in cardiac repair.

Upregulated miRNA	Model of Myocardial Injury	MSC-Exo/EV Source	Administration	Mechanisms/Signaling Pathway	Effects
miR-214	Rats MI by LAD ligation	Human Umbilical Cord	Intramyocardial	Negatively modulate PTEN and upregulate Akt signaling	Promote angiogenesis and preserve cardiac function
miR-129-5P	Mice MI by LAD ligation	bone marrow		Downregulate expression of HMGB1	Inhibit apoptosis and fibrosis; enhance cardiac function
miR-132	Mice MI by LAD ligation	Mouse bone marrow	Intramyocardial		Promote angiogenesis and preserve cardiac function
miR-19a	Rats MI by LAD ligation	Human Umbilical Cord		Downregulate expression of SOX6; activate AKT and inhibit JNK3/caspase-3 signaling activation	Inhibit apoptosis and preserve cardiac function
miR-301	Rats MI by LAD ligation	Rat bone marrow	Intramyocardial		Inhibit autophagy and enhance cardiac function
miR-146a	Rats MI by LAD ligation	Rat adipose tissue	Intravenous	Downregulate expression of EGR1 and inhibit TLR4/NFκB signaling activation	Inhibit apoptosis, inflammatory response, and fibrosis; reduce infarct size
miR-200-3b	Mice MI by LAD ligation		Intracardiac	Downregulate expression of BCL2L11 and NLRP1 signaling	Inhibit apoptosis and inflammatory response; reduce infraction size and improve cardiac function
miR-19a/19b	Mice Mi by ventilation-based method	Mouse bone marrow			Inhibit apoptosis and fibrosis; enhance the recovery of cardiac function
